# AI-enabled language technologies for language-mediated learning and clinical communication in international undergraduate dental education: a scoping review

**DOI:** 10.3389/fmed.2026.1921339

**Published:** 2026-09-03

**Authors:** Wenjuan Qiang, Xiaohong Deng, Tiezhou Hou, Le Qiang

**Affiliations:** 1 Key Laboratory of Shaanxi Province for Craniofacial Precision Medicine Research, College of Stomatology, Xi’an Jiaotong University, Xi’an, China; 2 Clinical Research Center of Shaanxi Province for Dental and Maxillofacial Diseases, College of Stomatology, Xi’an Jiaotong University, Xi’an, China; 3 Department of Cariology and Endodontics, College of Stomatology, Xi’an Jiaotong University, Xi’an, China

**Keywords:** artificial intelligence, clinical communication, dental education, generative artificial intelligence, international students, large language models, neural machine translation

## Abstract

**Background:**

International undergraduate dental students often learn and communicate in a language other than their first language, affecting terminology acquisition, clinical reasoning, patient explanations, informed consent, clinical communication, and patient safety. AI-enabled language technologies—including generative artificial intelligence (GenAI), large language models (LLMs), and neural machine translation (NMT)—may support these tasks, but their use in this population has not been systematically mapped.

**Methods:**

Following the Arksey and O'Malley framework, JBI guidance, and PRISMA-ScR, PubMed, Scopus, and Web of Science were searched from inception. Searches were conducted on 1 March 2026 and updated on 28 March 2026, supplemented by reference-list screening and targeted policy and grey-literature searches. Sources were charted by context, population, technology, outcomes, limitations, risks, and implementation, and classified by relevance.

**Results:**

Thirty-six non-policy sources and four policy or governance documents were included. Five involved undergraduate dental students, three addressed international, multilingual, or limited-English-proficiency learners or relevant policies, eight concerned other health-professions contexts, and eight were translation or technical benchmarks without learners. Categories overlapped, and only one source combined undergraduate dental students, an international or multilingual population, and an AI-enabled language technology. Applications included terminology translation, multilingual tutoring, communication rehearsal, and reflective-writing support. No source assessed retained learning, transfer to authentic clinical encounters, or patient-level outcomes.

**Conclusions:**

AI-enabled language technologies may support supervised language learning and patient-safety-oriented communication training, but direct evidence is sparse, short term, and largely derived from adjacent populations or technical benchmarks. Performance varies by language, task, prompt, model version, and context. Pending direct, comparative, longitudinal, and clinically situated evidence, implementation should include validated local resources, educator oversight, academic-integrity boundaries, privacy safeguards, and staged rehearsal before patient exposure.

## 1 Introduction

Dentistry is a communication-intensive profession in which learning, clinical reasoning and patient care are mediated by language. Undergraduate dental students must understand specialized anatomical, pathological and procedural terminology, and must also explain diagnoses, treatment options, risks and post-operative instructions in language that patients can understand (1, 2). Language is therefore not a peripheral educational issue; it is a central mechanism through which dental knowledge is acquired, expressed and translated into safe clinical interaction.

English-medium instruction (EMI) is widely used in internationally oriented health professions programs (3–5). As dental health professions education becomes increasingly internationalized, programs enroll increasingly diverse student populations. For learners studying in an additional language or with limited English proficiency (LEP), difficulty extends beyond cultural adjustment: they must learn, reason and communicate in high-stakes environments where communication failures can affect performance and patient care (6–9). Language processing may also increase cognitive burden and reduce the attention available for diagnostic reasoning and clinical decision-making (9). Because “international student”, “multilingual learner”, “non-native speaker” and “limited English proficiency” overlap but are not equivalent, Section 2.2 defines them separately.

AI-enabled language technologies are increasingly being explored as tools to support these learning and communication challenges. GenAI refers to artificial intelligence systems that can generate new content, including text, images, audio or code, by learning patterns from large datasets (10). LLMs are a type of GenAI trained on large amounts of text to understand, predict and generate human language (11). NMT systems are AI-based translation models that use neural networks to translate text or speech across languages by modelling sentences in context (12–16). Although these technologies differ technically— and NMT is not necessarily a form of generative AI — they all perform language-related functions that are relevant to international dental education; the umbrella term “AI-enabled language technologies” is therefore used throughout this review, with GenAI, LLMs and NMT distinguished wherever the distinction matters. Their usefulness is likely to vary by language. English is a high-resource language, meaning that large amounts of digital text, linguistic resources and technological support are available for model training and evaluation. Other languages, particularly regional or minority languages with fewer digital resources, may be less well represented in training data. This can increase the risk of inaccurate translation, fabricated information or poor performance in clinically specific communication (14, 17). These differences are important for dental education because international students may move between languages with very different levels of technological support.

Two recent scoping reviews provide broad but different foundations: El-Hakim et al. mapped AI applications, challenges and gaps across dental education (18), whereas Uribe et al. focused on the integration of GenAI and implementation recommendations in dental curricula (19). Neither review centered language mediation for international undergraduate dental students, explicitly separated technical performance from learner perceptions and clinical transfer, or graded each source by directness to that population–concept–context intersection. This review addresses that narrower gap by mapping terminology learning, multilingual tutoring, communication rehearsal, reflective writing and governance while keeping indirect evidence visibly distinct from direct evidence.

## 2 Methods

### 2.1 Study design, protocol and registration

A scoping review approach was chosen because the topic encompasses a broad and heterogeneous body of evidence, including empirical research, benchmarking studies, educational interventions, policy analyses and implementation-focused literature. The purpose was to map the extent, range and nature of the evidence rather than to estimate pooled intervention effects.

This review followed the five-stage framework of Arksey and O'Malley (20), refined through JBI guidance (21) and was reported using the Preferred Reporting Items for Systematic Reviews and Meta-Analyses extension for Scoping Reviews (PRISMA-ScR) (22); the completed PRISMA-ScR checklist is provided as Supplementary File S8. No formal *a priori* protocol document was prepared and the review was not registered; the review question, eligibility criteria and initial charting categories were agreed before screening, and the items added after piloting are reported in Section 2.6.

### 2.2 Review question, PCC framework and key concepts

The review question was: What is known about the use of AI-enabled language technologies to support language-mediated learning, communication equity and clinical communication among international undergraduate dental students? To improve transparency, the review scope was organized according to the Population–Concept–Context (PCC) framework shown in Table 1.

**Table 1:** The population-concept-context framework of this review.

PCC element	Operational definition in this review
Population	International undergraduate dental students; undergraduate dental students who are multilingual, study in an additional language or have LEP; and linguistically diverse dental/health-professions learners when transferability met Table 3
Concept	AI-enabled language technologies, including GenAI, LLMs, GPT-based chatbots, NMT and related language-oriented systems used for terminology translation, multilingual tutoring, communication rehearsal or language-focused academic support.
Context	Undergraduate dental education, clinical communication training, EMI/LEP learning environments and healthcare education governance contexts relevant to safe language-mediated communication.

Language-mediated learning was defined as the process through which students acquire, process and express disciplinary knowledge through language. For international students, this often involves moving between a first language (L1) and a second language (L2) during learning, clinical reasoning and patient communication.

The term “AI-enabled language technologies” was used as the umbrella term for the language-oriented technologies reviewed in this study, including GenAI systems, LLMs, GPT-based chatbots and NMT systems. NMT was included because it directly supports automated language mediation, although it is not necessarily a form of generative AI; NMT is therefore reported separately from GenAI and LLMs wherever the distinction is relevant. For clarity, four technical approaches are also distinguished consistently throughout the review: prompt engineering (structuring the user-facing input to the model), instruction configuration (system-level instructions that constrain model behavior), retrieval-augmented generation (RAG; connecting the model to a curated knowledge source at inference time) and fine-tuning (adapting model weights through additional training on domain data). The review included AI-enabled translation, multilingual tutoring, communication rehearsal and language-oriented reflective writing support. Sources focused exclusively on psychomotor simulation, such as haptic virtual reality devices, or on purely visual diagnostic AI applications, such as automated caries detection on radiographs, were excluded.

Because the population terms used in this field are related but not equivalent, and because different language-mediated tasks carry different educational needs and safety considerations, working definitions of the learner groups and task contexts considered in this review are provided in Table 2. International enrolment status was not used as a proxy for language difficulty: an international student may be fully proficient in the language of instruction, and a domestic student may study in an additional language. Sources were charted according to which learner group(s) and task context(s) they actually addressed.

**Table 2:** Working definitions of learner groups and language-mediated task contexts used in this review.

Category	Term/context	Working definition
Learner group	International student	A student enrolled in a degree program outside the country in which they completed prior education or hold citizenship; may or may not experience language difficulty.
	Non-native speaker	A student whose first language differs from the language of instruction, regardless of nationality or enrolment status.
	Multilingual learner	A student who uses two or more languages during learning; proficiency may be high in all languages used.
	Limited English proficiency (LEP)	A self-reported or measured limitation in English proficiency sufficient to affect safe and effective academic or clinical communication.
	English-medium instruction (EMI) student	A student taught through English in a setting where English is not the majority first language of the population or faculty.
Task context	Learning dentistry in a second language	Acquiring and expressing disciplinary knowledge in a language other than the learners first language.
	Communicating with patients in the host language	Patient-facing communication in the majority or institutional language of the clinical setting.
	Translating instructional content into the learner's first language	Using translation to access lectures, textbooks or terminology in the learners L1.
	Translating patient information into another language	Producing patient-facing material (e.g., instructions, consent information) in a language other than the source language.
	Communicating in a language non-native to both parties	Learner and patient interact in a lingua franca that is a second language for both, with compounded comprehension risks.

### 2.3 Eligibility criteria and directness classification

Sources were eligible if they addressed the use of AI-enabled language technologies, including GenAI, LLMs, GPT-based systems or NMT, to support language-mediated learning, multilingual communication, communication training or language-focused academic tasks in undergraduate dental education, or if they provided language-barrier, pedagogical or governance evidence necessary for interpreting that use. Because directly dental-specific evidence remains limited, studies from medical education, health professions education, patient-safety research and higher-education governance were also considered when they met the operational relevance rules in Table 3.

**Table 3:** Operational relevance rules and directness classification.

Decision question	Operational rule applied	Resulting classification
Was the population sufficiently relevant?	Undergraduate dental (or dental hygiene) students → eligible. Other undergraduate health-professions students, dental educators → eligible if the concept rules below were met. Postgraduates, clinicians, patients, general university students → eligible only under the conditions stated in the text.	Dental students → direct; other learners/educators → indirect; no learner population → contextual.
Did the study genuinely address language-mediated learning?	The source examined acquisition, processing or expression of disciplinary content in an additional language or across languages (e.g., translation, multilingual question answering, L2 writing), rather than digital learning within a single language only.	Determines eligibility; classification per population rule.
Was a communication or simulation study relevant?	The communication task involved patient-facing language functions (explanation, informed consent, risk communication, empathy) rather than purely psychomotor or procedural skills.	Determines eligibility; classification per population rule.
Were findings from medical education transferable to dentistry?	The language or communication function was profession-generic (e.g., terminology translation, patient instructions or OSCE-style communication), and interpretation was limited to communication processes rather than medicine-specific knowledge.	Indirect (learners present) or contextual (no learners).
Were benchmarking studies without learners eligible?	Eligible when the benchmarked language function mapped onto an educational task within the analytical framework (terminology translation, dental question answering, patient-facing instructions).	Contextual.
Was a governance document sufficiently relevant?	The document was issued by an international or supranational authority, formally published by the search date, and contained provisions applicable to AI-language use in education or to learner/patient data.	Contextual (governance).

Eligible sources included non-policy sources, technical benchmarks, evidence syntheses, educational interventions and relevant policy or governance documents. Records were excluded for the wrong population, concept or context (Table 3), an exclusive focus on visual diagnosis or psychomotor simulation, absence of empirical or directly relevant guidance content, or commentary/editorial format without empirical data or formal guidance. Evidence from postgraduate learners, clinicians or patients was retained only when the language/communication function was transferable to undergraduate teaching and was classified as indirect or contextual; it was not used to claim undergraduate learning effects. General higher-education sources were eligible only when they addressed academic integrity or writing support involving international or multilingual learners and were likewise treated as indirect/contextual evidence.

Each source was classified as directly relevant, indirectly relevant or contextual using Table 3. Two reviewers applied the classification independently during charting, resolved disagreements through joint review and discussion, and referred unresolved cases to a third reviewer.

Only English-language records were eligible. This restriction is somewhat paradoxical in a review concerned with multilingual and lower-resource language contexts, and it is discussed as an important limitation in Section 4.6.

### 2.4 Information sources and search strategy

PubMed, Scopus and Web of Science were searched from database inception. The initial searches were conducted on 1 March 2026 and were updated using the same conceptual structure on 28 March 2026. No publication-date restriction was applied. Reference lists of included sources were hand-searched.

Searches combined controlled vocabulary and free-text terms for AI-enabled language technologies, dental/health-professions education and language/communication. The following syntax illustrates the core search structure adapted for each database: (“artificial intelligence” OR “generative artificial intelligence” OR GenAI OR “large language model*” OR LLM* OR ChatGPT OR GPT OR “neural machine translation” OR “machine translation”) AND (“dental education” OR dentistry OR “dental student*” OR “undergraduate dental”) AND (“international student*” OR multilingual OR “limited English proficiency” OR “English-medium instruction” OR “language barrier*” OR communication OR “Objective Structured Clinical Examination” OR OSCE OR “virtual patient*” OR “reflective writing”). Retrospectively reconstructed database-specific strategies — including controlled vocabulary, free-text terms, field tags, filters, and the recorded execution and update dates, and the number of records retrieved per database — are reported in Supplementary Table S1. The exact line-by-line search histories and database-specific retrieval counts were not prospectively archived; consequently, minor differences between the reconstructed syntax and the searches originally executed cannot be excluded.

Policy and grey literature were sought on the websites of the World Health Organization, UNESCO, EUR-Lex and the European Data Protection Supervisor, supplemented by targeted Google searching. Concepts included artificial intelligence/generative AI, education, governance, data protection and health. Sixteen documents were screened in full and 4 met the stated relevance criteria. The grey-literature and policy search log is provided in Supplementary Table S2A, and full-text exclusion counts with their operational definitions are provided in Supplementary Table S2B.

Education-focused databases such as ERIC and PsycINFO were not searched. The database selection prioritized biomedical and multidisciplinary indexes because the review question centered on clinically situated language and communication functions in health-professions education, and because Scopus and Web of Science index the major education and educational-technology journals in which relevant studies of this type appear. Nevertheless, the omission of education-specific databases may have missed pedagogy-focused studies, and this is acknowledged as a limitation in Section 4.6.

### 2.5 Selection of sources of evidence

Records were imported into EndNote, and duplicates were removed using automated and manual checks. The deduplicated database set comprised 2,228 records. Two reviewers independently screened titles and abstracts and assessed full texts. Disagreements were resolved by joint rereading and application of the relevant eligibility rule; unresolved cases were referred to a third reviewer. Agreement was not formally quantified. Exclusions were coded as wrong concept, wrong source type, insufficient relevance or other; the five records in the residual “other” category were not prospectively subcategorized.

### 2.6 Data charting and domain development

**Figure 1:**
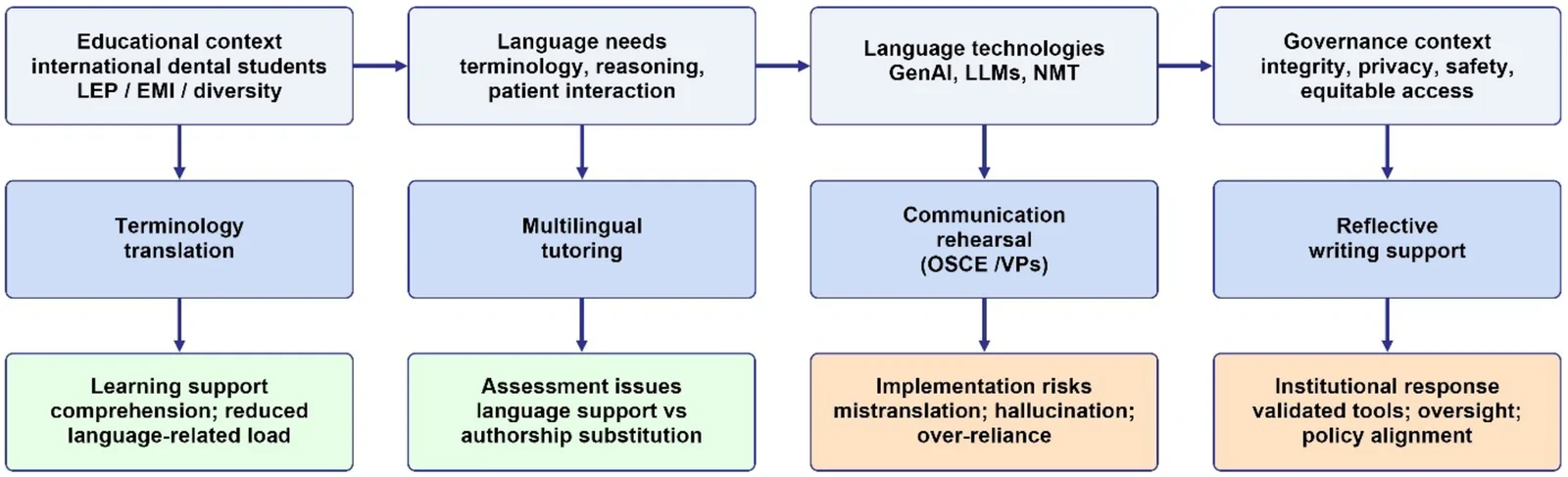
Analytical framework used to map AI-enabled language technologies for language-mediated learning, communication equity and clinical communication in international undergraduate dental education.

Data were charted using a structured form. The analytical framework in Figure 1 links the review scope, charting categories and synthesis domains. Initial items covered publication/source characteristics, context, target group, technology, language or communication function and outcome. Two reviewers piloted the form on 28 heterogeneous sources spanning benchmarks, interventions, surveys, reviews and policy documents. The pilot sample was deliberately large so that every source type in this heterogeneous set was represented, and all pilot sources that met the eligibility criteria remained in the final review. Piloting did not change eligibility; it added risk/implementation items, separated outcome levels and added the technical-reporting items in Section 2.7. The final framework is reproduced in Supplementary Table S6.

Two reviewers independently charted all included sources. They resolved differences through discussion and comparison with the source; unresolved items were referred to a third reviewer. Charting agreement was not formally quantified.

The four application domains were developed inductively by grouping sources according to their primary language or communication function: terminology translation; multilingual tutoring and knowledge support; communication rehearsal through OSCE-related tools and virtual patients; and reflective-writing support. Multi-function sources were assigned to a primary domain and cross-referenced narratively where relevant. Governance and implementation was treated consistently as a cross-cutting theme rather than a fifth application domain.

### 2.7 Critical appraisal and charting of methodological limitations

Consistent with JBI guidance, no formal critical appraisal with a scored quality-assessment instrument was undertaken, because the objective was to map the extent, range and nature of the literature rather than to grade the certainty of effect estimates. However, because this review informs recommendations concerning communication training, curriculum integration and governance, key methodological limitations of every included non-policy source, where applicable were charted and are reported descriptively. The charted items were: sample size; presence of a comparator; validation status of outcome measures; number and expertise of human evaluators; blinding and inter-rater reliability; number of model runs or repeated outputs; prompt disclosure; model name, version and date of access; proprietary or open-source status; prompting mode (zero-shot, few-shot or structured); system instructions or instruction configuration; generation settings where available; use of retrieval-augmented generation and the source and governance of retrieved content; fine-tuning or domain adaptation; human review or moderation of outputs; external or local validation; reporting of statistical uncertainty and confidence intervals; authenticity of the educational setting; and potential benchmark contamination.

For studies specifically evaluating LLM performance, charting was informed by applicable TRIPOD-LLM items. These items were not applied to NMT-only studies, qualitative research, educational interventions or policy documents when inappropriate. Source-level reporting is provided in Supplementary Table S5 and summarized in Section 3.6. “Not reported” in that table is not interpreted as evidence that a method was absent.

### 2.8 Synthesis of results

Findings were synthesized narratively, following the four application domains generated during charting. To avoid conflating different levels of evidence, outcomes were synthesized separately at six levels: (i) technical performance of the technology (e.g., translation accuracy, benchmark examination accuracy); (ii) learner perceptions (e.g., acceptability, confidence, perceived usefulness); (iii) short-term educational outcomes (e.g., knowledge-test performance immediately after use); (iv) assessed competence (e.g., examiner-rated communication performance); (v) behavioral transfer to authentic clinical encounters; and (vi) patient-related or clinical outcomes (e.g., patient comprehension, safety events). Cross-cutting issues related to evidence limitations, academic integrity, implementation and governance were synthesized across the four domains.

## 3 Results

### 3.1 Selection of sources of evidence

**Figure 2:**
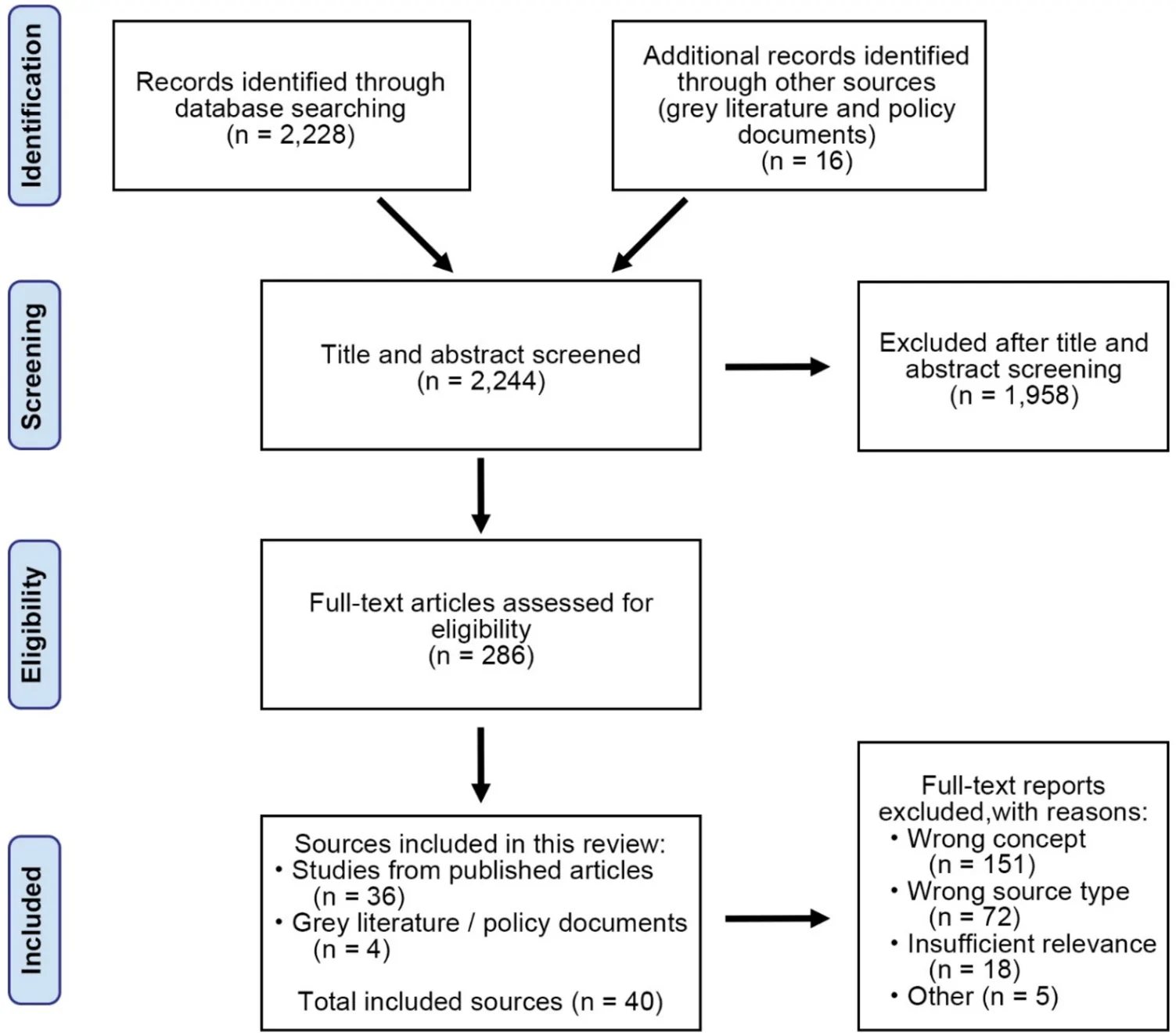
PRISMA-ScR flow diagram of the study-selection process.

After duplicate removal, 2,228 unique database records and 16 policy/grey-literature records were screened (*n* = 2,244). Title/abstract screening excluded 1,958 records, leaving 286 full-text reports. Of these, 246 were excluded: wrong concept (*n* = 151), wrong source type (*n* = 72), insufficient relevance (*n* = 18) and other reasons not prospectively subcategorized (*n* = 5). Forty sources were included: 36 non-policy research sources and 4 policy/governance documents. Figure 2 shows the selection process; Supplementary Table S2B reproduces the exclusion counts.

### 3.2 Overview and directness of the evidence base

The 36 non-policy sources spanned dental and medical education, translation, digital health, patient safety and higher-education governance. Table 4 applies the directness rules in Table 3. Five sources directly involved undergraduate dental student populations under the broad definition (23–27), but only Rath (27) combined dental undergraduates, an international/multilingual population and an AI-enabled language technology. Three sources involved international, multilingual or LEP populations (27–29); 8 drew on medical or other health-professions contexts (11, 12, 14, 30–34); 8 were translation or technical benchmarks without learners (12–16, 35–37); and 9 were other higher-education, language-barrier or pedagogical sources (9, 10, 38–44). Categories overlap and must not be summed.

**Table 4:** Directness of included evidence relative to the review question. Categories overlap.

Category	n	References
Broad directness: undergraduate dental students with a language, communication or AI-language focus	5	(23–27)
Full match: dental undergraduates + international/multilingual population + AI-enabled language technology	1	(27)
International, multilingual or LEP populations	3	(27–29)
Medical or other non-dental health-professions context	8	(11, 12, 14, 30–34)
Translation/technical benchmark without an educational learner population	8	(12–16, 35–37)
Other supporting source (dental-education review, educator survey, translation-methods survey or device-feasibility study) without an international or language-learner focus	6	(18–20, 45–47)
Other higher-education, language-barrier or pedagogy source	9	(9, 10, 38–44)
Policy or governance documents	4	(48–51)

Source-level classifications for all 36 sources are given in Supplementary Table S3A.

The single full-match source and the broader scarcity of direct evidence are principal findings. Accordingly, adjacent evidence is used to define plausible applications, risks and research priorities—not to claim demonstrated effects in international dental undergraduates.

Four inductively developed application domains were mapped: terminology translation; multilingual tutoring and knowledge support; communication rehearsal through OSCE-related tools and virtual patients; and reflective-writing support. Governance and implementation is cross-cutting. Table 5 maps the four domains.

**Table 5:** Evidence maps by application domain and charting category.

Application domain	Technology and source examples	Language or communication function	Main educational value	Key limitations or risks
Terminology translation	NMT systems, GPT-based translation tools and medical/dental terminology resources(12–16, 23, 27)	Translation of dental terminology, patient instructions and discipline-specific concepts across languages.	Supports comprehension of technical vocabulary and may reduce language-related learning load, particularly in high-resource language pairs.	Accuracy varies by language pair and clinical context; patient-facing or safety-related text requires human checking.
Multilingual tutoring and knowledge support	LLMs, GPT-4o, DeepSeek-R1, Claude, DentalBench and RAG-based dental systems (11, 35–37, 45)	Question answering, explanation of dental concepts and support for reasoning across languages.	May provide flexible learning support and help students practice disciplinary explanation in a second language.	Performance declines when input language, prompt language and reasoning language are misaligned; hallucination remains possible.
Communication rehearsal through OSCE-related tools and virtual patients	AI-generated OSCE stations, virtual patients, chatbots and communication-skill evaluation tools (25, 26, 31–34, 46, 47)	Practice of patient explanation, empathy, informed consent, risk communication and culturally appropriate phrasing.	Provides scalable, low-stakes rehearsal before real-patient encounters and may increase confidence for international students.	Requires clear scenario design, faculty oversight and validation against local assessment criteria.
Reflective writing support	GenAI writing support, reflective blogs and academic-integrity literature (28, 29, 38, 44)	Language polishing, sentence restructuring and expression of reflective clinical reasoning.	May help distinguish students’ clinical reasoning from second-language writing difficulty when used only for language support.	Boundary between language assistance and content generation must be explicitly governed.

Governance and implementation sources (18, 19, 28, 30, 48–51) are synthesized as a cross-cutting theme rather than as a fifth application domain.

### 3.3 Characteristics of included sources

Study-level characteristics of the 36 non-policy sources are presented in Supplementary Tables S3A,B, and the 4 policy/governance documents are presented separately in Supplementary Table S4.

### 3.4 Language and communication needs of international dental students

Across health professions education, foreign-language instruction has been recognized as a systemic learning barrier. This issue is particularly salient in dentistry because communication is integral to diagnosis, treatment explanation, informed consent and trust building (1, 2). Healthcare research has shown that language discordance and ineffective communication among patients with LEP are associated with adverse events, reduced comprehension and increased risk of harm (6–8, 41–43). These findings provide the theoretical patient-safety rationale for this review: they concern patient populations rather than dental students, and they do not demonstrate that student-facing language technologies reduce patient harm (see Section 4.4).

In clinical dental training, language difficulty is not limited to vocabulary recognition. It also affects the ability to explain reasoning, understand rapid clinical dialogue and communicate with patients and supervisors in real time. A mixed-methods study in dental education found that students had difficulty explaining procedures and disease mechanisms, including crown lengthening, periodontitis, prefabricated post and core, and indirect pulp capping (24). Such communication failures may reduce patient understanding, weaken adherence and undermine the ethical and legal basis of informed consent.

International dental students should not be treated as a homogeneous group. Needs vary with distance from the instructional language, prior clinical exposure, technology access and literacy, cultural expectations and institutional support. These factors and their implementation implications are summarized in Supplementary Table S7.

### 3.5 Applications of AI-enabled language technologies

#### 3.5.1 Terminology translation

Accurate understanding of technical terminology is a foundational requirement for dental learning. Benchmarking studies indicate that NMT systems and GPT-based tools can perform well for some forms of medical terminology translation, particularly in high-resource languages. For example, evaluation of Human Phenotype Ontology (HPO) term translation showed strong English-German performance for both GPT-3.5 and DeepL (15). Other comparative work suggests that machine translation may approach professional-level output in some language pairs, while performance is weaker in languages or contexts with fewer digital resources (12).

These findings do not justify uncritical adoption. GPT-based systems may generate fluent output while still changing meaning, and performance varies across languages and clinical contexts (12–16). Patient-facing translations have included clinically significant errors (12, 14). Terminology translation may therefore support learning, but clinically sensitive content requires educator or clinician review. The single full-match study belongs to this domain. In an eight-week convergent mixed-methods pilot, 35 Chinese international Year 1–2 dental students used ChatGPT (GPT-4) for terminology learning and concept explanation in English-medium oral anatomy; mean comprehension increased by 5.7 points (*p* < 0.001, *d* = 1.91), with positive engagement themes. As an uncontrolled, single-site pilot with self-report components, this constitutes short-term educational-outcome evidence at best. The remaining evidence in this domain sits at the technical-performance level: none of the translation sources measured dental-student learning, and technical reporting was incomplete in several sources (Supplementary Table S5).

#### 3.5.2 Multilingual tutoring and knowledge support

LLMs may support question answering, concept explanation and adaptive tutoring for international students, but their performance is strongly language-dependent. In medical education, LLMs have been benchmarked against examinations such as the United States Medical Licensing Examination (USMLE) and specialty question banks (11, 36). DentalBench, a cross-lingual benchmarking study using a bilingual Chinese-English dental question bank, showed that high-performing systems such as DeepSeek-R1, GPT-4o and Claude can achieve high accuracy on professional dental questions, but mainly under favorable language-alignment conditions (35, 37).

Cross-linguistic studies show that performance can change with question language, prompt language and model (11). Domain grounding through retrieval-augmented generation may improve relevance in a defined setting, but the dentistry literature is still largely conceptual (45). Direct dental-student evidence in this domain comes from a single randomized controlled study in which 59 dental students prepared for a 25-item dental-trauma knowledge test using ChatGPT-4o, a digital textbook, a dedicated app or no support. All three supported groups outperformed the control; ChatGPT-4o accuracy (about 86.4%) was comparable to the textbook and the app, completion was faster than with the textbook, and clearer student prompts were associated with clearer responses and correct answers (12). This is a short-term knowledge outcome from one center, without an international or LEP subgroup. Static-question accuracy demonstrates model performance, not tutoring effectiveness: no included study showed that LLM tutoring improved assessed dental competence, and public benchmarks may be vulnerable to contamination (36, 37).

#### 3.5.3 Communication rehearsal through OSCE-related tools and virtual patients

The Objective Structured Clinical Examination (OSCE) is commonly used to assess communication and clinical reasoning in medical and dental education (46). For international students, OSCEs can be difficult because they require real-time language organization, empathic expression and culturally appropriate communication. Repeated practice with standardized patients is educationally valuable, but it is often difficult to provide at scale because it requires trained personnel, scheduling and financial resources.

Generative LLMs may assist OSCE preparation by helping educators draft stations and rubrics for expert review (33) or by providing supervised virtual-patient rehearsal (25, 26, 31, 32). Reported outcomes were chiefly feasibility, usability, satisfaction and confidence, with limited competence-level assessment. Confidence should not be equated with communication competence, and no included source followed learners from AI rehearsal into authentic patient encounters.

#### 3.5.4 Reflective writing support

Reflective practice is an important component of advanced dental education, but international students may struggle to express clinical insight in clear academic English (44). GenAI tools may support vocabulary refinement, sentence restructuring and professional tone adjustment after students have first produced their own clinical reasoning (29). Used in this restricted way, language technologies may help educators assess the quality of students' reasoning without over-penalizing second-language writing difficulty.

The same tools also create academic-integrity concerns. The central distinction is between language support, such as grammar correction or clearer phrasing, and authorship substitution, in which the tool generates the substantive ideas or reflection on behalf of the student. This distinction needs to be stated explicitly in assessment instructions and institutional policies. Evidence in this domain consists mainly of learner perceptions, policy analyses and pre-AI pedagogical research; no included study measured the effect of AI-supported reflective writing on assessed reflective competence.

#### 3.5.5 Outcome levels across domains

Across domains, outcomes clustered at technical performance and learner perception, with few short-term educational outcomes. No included source assessed retained learning, independent communication after AI withdrawal, behavioral transfer to authentic clinical encounters, or patient comprehension, experience or safety outcomes.

### 3.6 Methodological characteristics and limitations of the included studies

Source-level methodological reporting (Supplementary Table S5) shows recurrent limitations: small or convenience samples in learner studies; heterogeneous and sometimes unvalidated outcomes; incomplete reporting of prompts, model versions, access dates, generation settings and repeated runs; variable description of evaluators, blinding and agreement; public-item benchmark contamination risk; and few authentic, longitudinal learner evaluations. A pooled median sample size or single denominator across all 36 non-policy sources would be misleading because the set combines reviews, policy analyses, technical benchmarks without participants and learner studies. “Not reported” is therefore shown at source level rather than counted as “No”.

## 4 Discussion

### 4.1 Principal findings

This scoping review suggests that AI-enabled language technologies may support international undergraduate dental students by reducing language-related barriers at several points in the learning pathway. Their strongest potential lies in terminology translation, multilingual explanation of dental concepts, repeated communication rehearsal and language-focused support for reflective writing. These technologies should be understood as scaffolds for language-mediated learning, not as replacements for disciplinary knowledge, clinical judgement or supervised patient communication.

Two findings frame the discussion. First, only one source fully combined undergraduate dental students, an international/multilingual population and an AI-enabled language technology (27); the remaining evidence is direct only under broader criteria or is indirect/contextual (Table 4). Second, performance and usefulness vary with language, task, model/version, prompt, clinical context, local resources and oversight. The discussion therefore distinguishes mapped possibilities from demonstrated educational effects.

### 4.2 Interpreting different levels of outcomes

Because the included sources measured different outcome levels (Section 2.8), three conflations must be avoided. Examination accuracy is not tutoring effectiveness; confidence is not competence; and fluent language is not necessarily clinically accurate (12, 14). Whether these applications produce retained learning, independent communication, transfer to authentic encounters or improved patient outcomes remains untested in the included evidence. These distinctions set the future-research priorities in Section 4.7.

### 4.3 Academic integrity and assessment implications

Academic integrity is one of the most sensitive issues in the adoption of GenAI and LLMs. International students may use these tools for translation, grammar correction and idea organization, while other students may use the same tools to generate content. Treating all AI-assisted text as equivalent misconduct risks obscuring the difference between permissible language assistance and unacceptable authorship substitution (28, 38).

More equitable assessment policies should define acceptable and unacceptable uses of AI-enabled language technologies. For example, grammar correction, terminology checking and translation of student-generated text may be permissible when declared, whereas generating clinical reasoning, reflective content or answers to assessment tasks may not be. Dental schools may also need to place greater emphasis on supervised assessments such as OSCEs, oral examinations and real-time reasoning tasks, where students demonstrate their own understanding and communication ability.

### 4.4 Implementation, governance, patient safety and equity

Educational value alone is not sufficient for safe implementation. Dental schools require governance pathways covering tool selection, validation, privacy, access equity, faculty development, curriculum integration and ongoing monitoring; governance is treated here as a cross-cutting theme that applies to all four application domains (18, 19, 28, 30, 48–51). Human-in-the-loop oversight should remain a baseline requirement, meaning that educators or clinicians review outputs before they are used in clinically sensitive teaching or patient-facing contexts (30).

Local validation is also important. Institution-approved terminology lists, bilingual glossaries, translated case examples and expert-reviewed communication scripts can help reduce the risk of inaccurate translation or misleading explanation. These resources are especially valuable when students work across high-resource and lower-resource languages, where model performance may be less predictable (14, 17).

Four levels must remain distinct: (i) the patient-safety rationale from language-barrier evidence (6–8, 41–43); (ii) technical language performance (13–16, 23); (iii) learner communication behavior; and (iv) patient comprehension or safety outcomes. Included sources support levels (i) and (ii), offer limited perception-level evidence relevant to (iii), and provide no evidence at level (iv). The manuscript therefore uses “patient-safety-oriented communication training” and does not claim reduced harm.

Similarly, communication equity cannot be inferred from access to translation or language support alone. Equity also depends on the cost of tools, technology access, digital literacy, privacy protections, the representation of learners' languages in training data, disability access and differential model performance across languages and dialects (17, 49–51). Free or institutionally provided access to a tool that performs poorly in a student's language may create an illusion of support while leaving the underlying inequity in place.

### 4.5 Implications for dental schools

**Figure 3:**
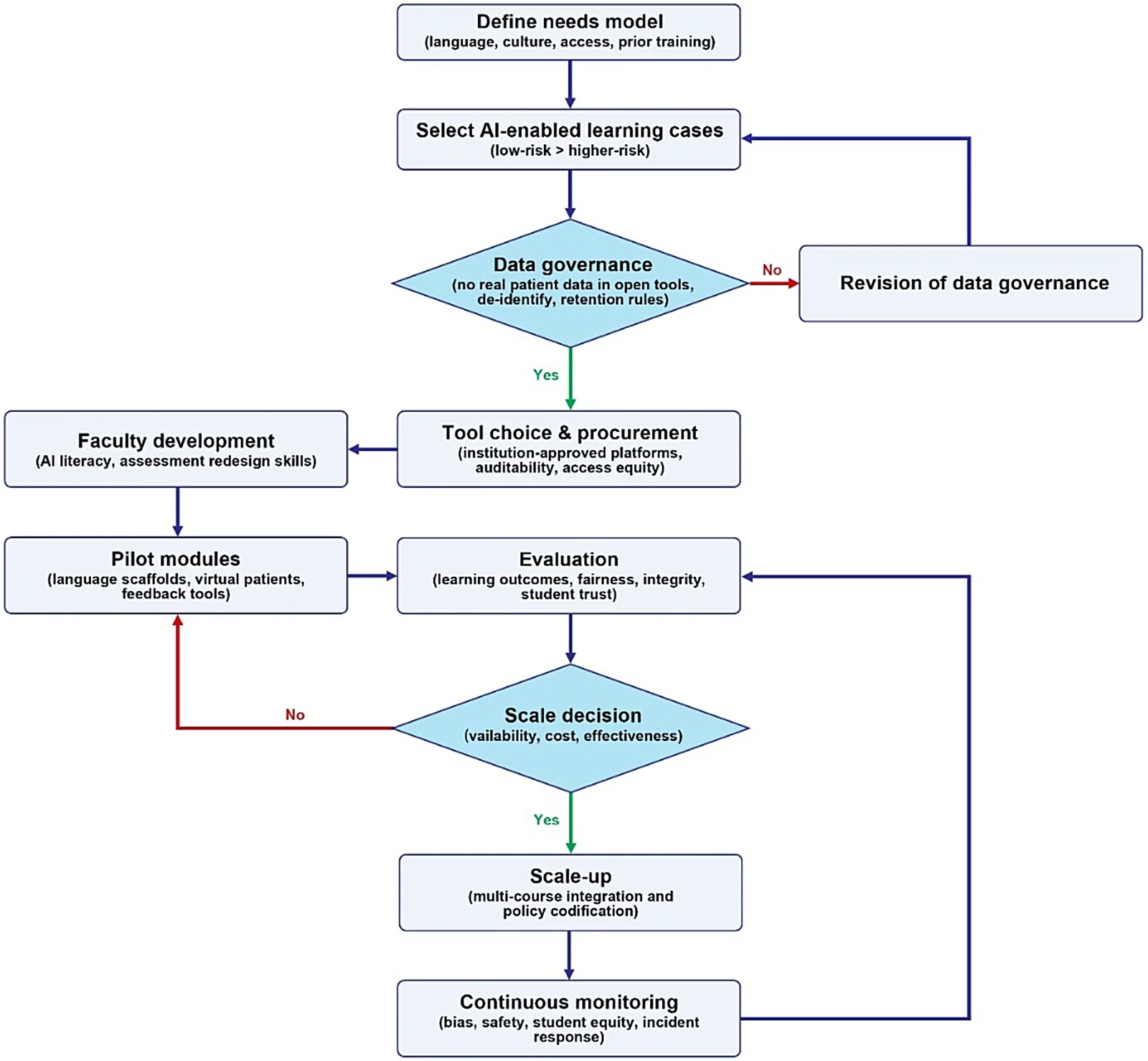
Responsible integration of AI-enabled language technologies in international undergraduate dental education: an author-developed conceptual pathway (not empirically validated).

For dental schools, the practical issue is how—not simply whether—to integrate these tools. Priorities include AI-language literacy, validated terminology resources, explicit academic-integrity rules, privacy safeguards and staged supervised rehearsal (19, 47). Table 6 links each recommendation to empirical, policy or author-derived support. Figure 3 is an author-developed implementation pathway synthesized from the mapped evidence and has not been validated by consensus or empirical testing.

**Table 6:** Recommendations and their evidentiary basis.

Recommendation	Evidentiary basis	Basis type
Use institutionally validated terminology resources and bilingual glossaries, with human checking of clinically sensitive translations.	Empirical evidence of variable translation accuracy across language pairs and of clinically significant mistranslations (12–15, 17); human-in-the-loop translation workflows (30)	Empirical (technical-performance level) + author-proposed extension to dentistry
Maintain educator (human-in-the-loop) review before AI outputs are used in clinically sensitive teaching or patient-facing contexts.	Empirical workflow evaluation (30); governance principles (48, 49)	Empirical + policy
Define permissible language support vs. authorship substitution explicitly in assessment policy.	Empirical and policy-analysis evidence on integrity and international learners (28, 29, 38); policy guidance (49)	Empirical (perception/policy-analysis level) + policy
Provide staged, supervised communication rehearsal with AI virtual patients before real-patient exposure.	Feasibility- and perception-level studies of virtual patients and AI-supported OSCE tools (25, 26, 31–33)	Empirical (feasibility/perception level) + author-proposed sequencing
Apply data-protection and privacy safeguards to student and patient data used with these tools.	Policy and regulatory documents (50, 51)	Policy
Embed AI language literacy in competency frameworks and faculty development.	Dental-education reviews and educator survey (18, 19, 47); policy guidance (49)	Indirect empirical + policy + author-proposed
Adopt the institutional implementation pathway (Figure 3).	Synthesized from the rows above.	Author-developed conceptual framework (not validated)

### 4.6 Strengths and limitations of this review

Strengths include independent dual screening and charting, explicit PCC/directness rules and separation of outcome levels. Limitations are substantial. Only English-language records were eligible, potentially excluding relevant multilingual and lower-resource-language evidence. ERIC and PsycINFO were not searched, so pedagogy-focused work may have been missed. The grey-literature search was targeted rather than exhaustive. The exact line-by-line database search histories and database-specific retrieval counts were not prospectively archived; the strategies in Supplementary Table S1 were reconstructed retrospectively and may differ slightly from the searches originally executed. The five full-text exclusions coded as “Other” were not prospectively subcategorized, so a more granular *post hoc* breakdown was not possible. No formal *a priori* protocol was prepared or registered; therefore, protocol deviations were not assessed. No scored critical appraisal was undertaken; limitations were charted descriptively. Much evidence is indirect, and screening/charting agreement was not formally quantified. Finally, rapid model change limits the durability of model-specific findings.

### 4.7 Limitations of the current evidence base and future research

The current evidence base has several limitations. First, many benchmark studies assess competence using static text-based questions, whereas authentic dental communication involves speech, images, facial expression, clinical context and interpersonal judgement (36). Second, translation and tutoring performance remain uneven across languages, particularly when languages have fewer digital resources or when clinical language is culturally specific (14, 17). Third, current systems may miss indirect meanings, emotionally sensitive statements and culturally specific communication cues that are central to informed consent and patient-centered care.

Over-reliance is another concern. Excessive dependence on AI-supported translation or expression may weaken students' independent communication ability and encourage automation bias, where learners accept machine output too readily. Future research should therefore prioritize comparative and longitudinal designs involving international undergraduate dental students; evaluate retained learning and students' independent communication ability after AI support is withdrawn; test transfer from AI-based rehearsal to real clinical encounters, including patient comprehension and experience; develop assessments that combine text, speech and visual information across languages; examine performance for regional, minority and under-represented languages; and report LLM evaluations in accordance with TRIPOD-LLM (52), including model versions, access dates, prompts and repeated runs.

## 5 Conclusions

This scoping review mapped AI-enabled language technologies for language-mediated learning and clinical communication in international undergraduate dental education. Four domains emerged: terminology translation, multilingual tutoring and knowledge support, communication rehearsal, and reflective-writing support. Only one source fully matched the target population, concept and context; much of the evidence is indirect or technical, performance varies with language, task, prompt, model version and context; and no included source demonstrated retained learning, authentic clinical transfer or patient benefit. Dental schools should therefore treat these tools as supervised supports, not autonomous solutions, and pair them with validated local resources, clear integrity rules, educator review, privacy safeguards and staged communication practice while direct longitudinal evidence develops.
