# Supplementary material for: AI-enabled language technologies for language-mediated learning and clinical communication in international undergraduate dental education: a scoping review
Source: Front Med (Lausanne). 2026 Sep 3;13:1921339. doi: 10.3389/fmed.2026.1921339 (PMC13581666; doi:10.3389/fmed.2026.1921339)
Supplement: Supplementary file 1 [file Datasheet1.docx]

**Supplementary Materials**

**AI-Enabled Language Technologies for Language-Mediated Learning and Clinical Communication in International Undergraduate Dental Education: A Scoping Review**

**Author**

Wenjuan Qiang^1, 2, 3^, Xiaohong Deng^1, 2, 3^, Tiezhou Hou^1, 2, 3 *^ and Le Qiang^1, 2, 3 *^

1. Key laboratory of Shaanxi Province for Craniofacial Precision Medicine Research, College of Stomatology, Xi’an Jiaotong University

2. Clinical Research Center of Shaanxi Province for Dental and Maxillofacial Diseases, College of Stomatology, Xi’an Jiaotong University

3. Department of Cariology and Endodontics, College of Stomatology, Xi’an Jiaotong University

Corresponding author:

Tiezhou Hou, tiezhou@mail.xjtu.edu.cn;

Le Qiang, qiangle@xjtu.edu.cn

**Contents**

Table S1. Retrospectively reconstructed database-specific search strategies ......................................................... 2

Table S2A. Grey-literature and policy search log ................................................................................................... 3

Table S2B. Full-text exclusion counts and operational exclusion criteria .............................................................. 3

Tables S3A–S3B. Characteristics and findings of the 36 non-policy sources ........................................................ 4

Table S4. Included policy and governance documents .......................................................................................... 15

Table S5. Methodological reporting and limitations of the included non-policy sources ..................................... 15

Table S6. Operationalization of the data-charting framework ............................................................................... 20

Table S7. Learner and institutional factors relevant to AI-enabled language support ........................................... 21

Table S8. Completed PRISMA-ScR checklist with manuscript locations ............................................................ 22

References ............................................................................................................................................................ 23

**Supplementary Table S1. Retrospectively reconstructed database-specific search strategies**

| **Database** | **Retrospectively reconstructed search strategy** | **Coverage/filters** | **Execution dates** |
| --- | --- | --- | --- |
| PubMed | (("Artificial Intelligence"[Mesh] OR "artificial intelligence"[tiab] OR "generative artificial intelligence"[tiab] OR GenAI[tiab] OR "large language model*"[tiab] OR LLM*[tiab] OR ChatGPT[tiab] OR GPT[tiab] OR "neural machine translation"[tiab] OR "machine translation"[tiab]) AND ("Education, Dental"[Mesh] OR "dental education"[tiab] OR dentistry[tiab] OR "dental student*"[tiab] OR "undergraduate dental"[tiab]) AND ("international student*"[tiab] OR multilingual[tiab] OR "limited English proficiency"[tiab] OR "English-medium instruction"[tiab] OR "language barrier*"[tiab] OR communication[tiab] OR "Objective Structured Clinical Examination"[tiab] OR OSCE[tiab] OR "virtual patient*"[tiab] OR "reflective writing"[tiab])) | From database inception; English-language eligibility applied during screening; no publication-date filter | Initial: 01 March 2026; update: 28 March 2026 |
| Scopus | TITLE-ABS-KEY(("artificial intelligence" OR "generative artificial intelligence" OR GenAI OR "large language model*" OR LLM* OR ChatGPT OR GPT OR "neural machine translation" OR "machine translation") AND ("dental education" OR dentistry OR "dental student*" OR "undergraduate dental") AND ("international student*" OR multilingual OR "limited English proficiency" OR "English-medium instruction" OR "language barrier*" OR communication OR OSCE OR "virtual patient*" OR "reflective writing")) |  |  |
| Web of Science Core Collection | TS=(("artificial intelligence" OR "generative artificial intelligence" OR GenAI OR "large language model*" OR LLM* OR ChatGPT OR GPT OR "neural machine translation" OR "machine translation") AND ("dental education" OR dentistry OR "dental student*" OR "undergraduate dental") AND ("international student*" OR multilingual OR "limited English proficiency" OR "English-medium instruction" OR "language barrier*" OR communication OR OSCE OR "virtual patient*" OR "reflective writing")) |  |  |

*Note: The exact line-by-line search histories and database-specific retrieval counts were not prospectively archived. The strategies shown above were reconstructed retrospectively from the review team’s recorded concepts, databases, field structures, filters and dates; minor differences from the searches originally executed cannot be excluded. This limits exact reproducibility. The PRISMA diagram reports 2,228 unique database records after deduplication and 16 policy or grey-literature records, yielding 2,244 records for screening.*

**Supplementary Table S2A. Grey-literature and policy search log**

| **Issuer/site** | **Platform** | **Concepts used** | **Date** | **Included document** |
| --- | --- | --- | --- | --- |
| World Health Organization | WHO website | artificial intelligence; ethics; governance; health | 1 March 2026 | Ethics and governance of artificial intelligence for health[1] |
| UNESCO | UNESCO website | generative AI; education; research; guidance | 1 March 2026 | Guidance for generative AI in education and research[2] |
| European Union | EUR-Lex | artificial intelligence act; education; vocational training; transparency | 1 March 2026 | Regulation (EU) 2024/1689[3] |
| European Data Protection Supervisor | EDPS website | generative AI; EUDPR; data protection; compliance | 1 March 2026 | Generative AI and the EUDPR[4] |
| Supplementary web search | Google | site/phrase combinations using AI, education, governance, data protection and health | 1 March 2026 | No additional included document reported |

Sixteen policy or grey-literature documents were screened in full, and four were included.

**Supplementary Table S2B. Full-text exclusion counts**

| **Reason** | **n** | **Operational description** |
| --- | --- | --- |
| **Wrong concept** | 151 | Did not address AI-enabled language technology, language/communication function or the operationally relevant contextual evidence. |
| **Wrong source type** | 72 | Commentary/editorial without empirical data or formal guidance, or another ineligible source type. |
| **Insufficient relevance** | 18 | Did not meet the direct, indirect or contextual relevance rules in Table 3. |
| **Other** | 5 | Residual record-specific eligibility reasons not captured by the three categories above; not prospectively subcategorized. |
| **Total excluded after full-text assessment** | 246 | 151 + 72 + 18 + 5 = 246. |

*Note: The five records coded as ‘Other’ were not prospectively subcategorized; reliable post hoc specification was therefore not possible.*

**Supplementary Table S3A. Characteristics of the 36 non-policy sources: context and population**

Directness categories follow Table 3 of the manuscript. ‘Undergraduate dental population’ under the broad definition is not equivalent to a full match with the international/multilingual AI-language review question.

| **Source** | **Country/**  **setting** | **Design** | **Participant group/sample** | **Dental/international/multilingual** | **Directness** |
| --- | --- | --- | --- | --- | --- |
| Hamad et al. (2025)[5] | Multi-country | Systematic review | 49 studies; >14,500 health-professions students | Dental: no; language-barrier focus: yes | Contextual |
| Bittle & El-Gayar (2025) [6] | USA | Systematic review | Higher-education studies (review level) | Dental: no; intl.: partial | Contextual |
| Strasser et al. (2026)[7] | Multilingual medical benchmark | Mixed-methods benchmarking | No learner population; 114 public medical MCQs | Dental: no | Contextual |
| Brewster et al. (2024)[8] | USA | Comparative translation benchmark | No learner population; 20 pediatric discharge instructions | Dental: no | Contextual |
| Haupt et al. (2025)[9] | Germany | Randomized controlled study | 59 dental students: ChatGPT-4o 15; textbook 15; AcciDent app 15; control 14 | Dental: yes; intl.: partial | Direct (dental population); not language-specific |
| Hendy et al. (2023)[10] | N/A | Benchmarking (preprint) | No learner population | Dental: no | Contextual |
| Kong et al. (2026)[11] | USA | Comparative accuracy/safety evaluation | No learner population | Dental: no | Contextual |
| Noll et al. (2025)[12] | Germany | Comparative terminology benchmark | No learner population; 120 Human Phenotype Ontology terms | Dental: no | Contextual |
| Sun (2024)[13] | USA | Linguistic translation evaluation | No learner population | Dental: no | Contextual |
| Ranathunga et al. (2023)[14] | N/A | Survey (methodological review) | No learner population | Dental: no | Contextual |
| El-Hakim et al. (2025)[15] | Multi-country | Scoping review | Dental-education studies (review level) | Dental: yes; intl.: no | Indirect |
| Uribe et al. (2025)[16] | Multi-country | Scoping review | Dental-education studies (review level) | Dental: yes; intl.: no | Indirect |
| Rath (2025)[17] | Malaysia (SEGi University) | Convergent mixed-methods pilot; 8 weeks | 35 Chinese international Year 1–2 dental students | Dental: yes; multilingual: yes | Direct; only source matching dental + international/multilingual + AI-language criteria |
| Cai et al. (2025)[18] | Preprint/model development | Multimodal model development and benchmark (preprint) | No learner population; approximately 120,000 dental images | Dental domain only | Contextual |
| Firoozi et al. (2025)[19] | N/A | Mini-review | No learner population | Dental domain only | Contextual |
| Wang Q. et al. (2026)[20] | N/A | Review of benchmarks | No learner population | Dental: no | Contextual |
| Zhu et al. (2025)[21] | China/preprint | Bilingual benchmark (preprint) | No learner population; 36,597 English–Chinese questions across 4 tasks and 16 dental subfields | Dental domain only | Contextual |
| Jones et al. (2025)[22] | Australia | Classroom implementation/usability study | Approximately 20 dental students; usability survey n=14 | Dental: yes; intl.: partial | Direct (dental population); not international/language-specific |
| Katonai et al. (2025)[23] | N/A | Scoping review | No learner population | Dental: no | Contextual |
| Liu et al. (2025)[24] | Chinese Taiwan | Feasibility/device evaluation | 30 dental interns | Dental: yes; intl.: partial | Indirect/contextual |
| Lopez-Garcia et al. (2026)[25] | USA | LLM evaluation study | Clinician–patient encounters; no students | Dental: no | Contextual |
| Suárez et al. (2022)[26] | Spain | Descriptive cross-sectional educational study | 193 fourth- and fifth-year dental students | Dental: yes; intl.: partial | Direct (dental population); not international/language-specific |
| Uribe et al. (2024)[27] | Multi-country | Cross-sectional worldwide survey; 31 items (May–June 2023) | 428 dental educators; reported response rate 28% | Educators (not students) | Indirect |
| Wang L. et al. (2024)[28] | China | Evaluation study | Resuscitation-examination candidates | Dental: no | Contextual |
| Zouakia et al. (2026)[29] | France | Comparative configuration study | No student participants; 24 generated OSCE stations across 8 topics | Dental: no | Indirect/contextual |
| Bannister et al. (2024)[30] | 11 countries | Qualitative document/policy analysis | 131 higher-education policies; international-student policy focus | Intl.: yes; dental: no | Indirect |
| Kofinas et al. (2025)[31] | UK | Exploratory experimental marking and moderation study | Higher-education students | Dental: no | Contextual |
| Sterling et al. (2025)[32] | Australia | Structured exercise-based educational study | International students | Intl.: yes; dental: no | Indirect |
| Wetmore et al. (2010)[33] | USA | Controlled two-group pre/post educational study (pre-AI) | 58 first-year dental-hygiene students: intervention 28; control 30 | Dental hygiene; no AI | Contextual |
| Brewster et al. (2025)[34] | USA | Multidisciplinary evaluation | Clinician reviewers; no students | Dental: no | Contextual |
| Amano & Berdejo-Espinola (2025)[35] | N/A | Review/perspective | No learner population | Dental: no | Contextual |
| Ross & Stuckler (2025)[36] | Multi-country review | Systematic review | University EFL students (review level) | Dental: no | Contextual |
| Al Shamsi et al. (2020)[37] | Multi-country | Systematic review | Patients / health services (review level) | Dental: no | Contextual |
| Saigal & Lewis (2023)[38] | USA | Book chapter (case-based) | N/A | Dental: no | Contextual |
| Sliwinski et al. (2024)[39] | USA | Review | Hospitalized LEP patients (review level) | Dental: no | Contextual |
| Khanagar et al. (2026)[40] | Saudi Arabia (Riyadh) | Mixed-methods study | 40 dental students and 40 patients | Dental: yes; language-barrier focus: yes | Direct (dental population); no AI concept |

**Supplementary Table S3B. Characteristics of the 36 non-policy sources: technology, task and findings**

| **Source** | **Language(s)** | **Technology** | **Task/comparator** | **Outcome measures** | **Main findings** | **Principal limitations** |
| --- | --- | --- | --- | --- | --- | --- |
| Hamad et al. (2025)[5] | Multiple foreign languages used for instruction | None (language-barrier evidence; no AI) | Educational barriers under foreign-language instruction | Barrier themes and educational outcomes | Foreign-language instruction created recurrent comprehension, participation and assessment barriers; marked heterogeneity across settings. | Review-level and non-dental evidence; no AI intervention. |
| Bittle & El-Gayar (2025) [6] | N/A | GenAI (general) | Academic integrity in higher education | Thematic categories of GenAI use, benefits and academic-integrity risks across 41 included studies | GenAI can enhance educational engagement and efficiency but also creates substantial risk of academic dishonesty; the authors recommend strengthening digital literacy and developing more robust detection approaches. | Review-level, non-dental and non-clinical evidence; four business/computing databases with the last search in May 2024; no measured learner outcomes. |
| Strasser et al. (2026)[7] | German, French and Italian | LLMs from OpenAI, Meta, Anthropic and DeepSeek | Multilingual medical MCQ answering across models, prompts and languages; comparator: Across languages and models | Answer accuracy and qualitative error patterns | Performance varied by model, prompt and language alignment. | No learners; public-item benchmark; exact prompts/runs require full-text checking. |
| Brewster et al. (2024)[8] | English to Spanish, Brazilian Portuguese and Haitian Creole | ChatGPT-4.0 and Google Translate | Patient-facing discharge-instruction translation; professional translation comparator; comparator: ChatGPT vs Google Translate | Adequacy, fluency, preservation of meaning and error severity | Quality varied by language; Haitian Creole output included clinically concerning errors. | Small corpus; pediatric and non-dental context; no educational outcomes. |
| Haupt et al. (2025)[9] | German | ChatGPT-4o | Emergency management of dental trauma; 25 dichotomous knowledge questions; comparator: Control condition | Correct-answer percentage, completion time and prompt clarity | ChatGPT-supported accuracy was about 86.4%; prompt clarity was associated with performance. | Single-center, short-term test; no international/LEP subgroup or clinical transfer. |
| Hendy et al. (2023)[10] | 18 translation directions covering high- and low-resource languages and non-English-centric pairs | GPT models versions | General and domain machine translation quality; comparator: GPT vs commercial MT systems | Automatic and human MT metrics | GPT models achieved competitive translation quality for high-resource languages but were markedly weaker for low-resource languages; prompting strategy affected performance, and hybrid GPT–NMT approaches outperformed either system alone. | Preprint; no learner population |
| Kong et al. (2026)[11] | English into Spanish, Chinese and Russian | MT systems | Translation of patient-specific discharge instructions; comparator: Across systems/languages | Sentence- and instruction-set-level translation accuracy and potential for clinical harm, coded by physicians from professional back-translations of 50 instruction sets (316 sentences) | Accuracy varied by language pair; some clinically significant mistranslations | Emergency-department discharge instructions rather than dental education; no learner population; only three target languages; harm judged from back-translations rather than patient comprehension. |
| Noll et al. (2025)[12] | English–German | GPT-3.5 and DeepL | Medical terminology translation with expert review; comparator: GPT-3.5 vs DeepL | Terminology-translation accuracy | Both systems showed strong English–German performance. | Terminology-only corpus; no dental learners or educational outcomes. |
| Sun (2024)[13] | Chinese–English | ChatGPT; DeepL | Translation of implied subjects; comparator: ChatGPT vs DeepL | Qualitative comparison of how implied subjects were rendered, with manual error analysis of a literary Chinese–English corpus | ChatGPT inferred implied subjects more accurately and produced more concise and readable output, whereas DeepL translated more literally to reduce potential misunderstanding. | Single literary (non-medical) source text; qualitative analysis without automatic metrics; model versions not specified; no clinical or educational outcome. |
| Ranathunga et al. (2023)[14] | Low-resource languages | NMT (methods survey) | Low-resource machine translation methods | N/A | Synthesizes NMT techniques for low-resource pairs (data augmentation, multilingual NMT, unsupervised NMT, pivoting, transfer learning) and provides guidance on selecting a technique for a given data setting; performance remains below high-resource pairs because parallel corpora are scarce. | Methodological survey without primary evaluation; no clinical, educational or health-communication data. |
| El-Hakim et al. (2025)[15] | N/A | AI (broad) | AI applications in dental education | N/A | Seventeen studies across four domains (preclinical training; clinical, diagnostic and radiographic training; assessment and feedback; content generation). AI improved procedural accuracy, diagnostic confidence, assessment efficiency and content delivery, but performed poorly for nuanced competencies such as manual dexterity and clinical judgement. | Review-level evidence from 17 heterogeneous studies (six databases, searched March 2025); English-language studies only; no focus on language, communication or international learners. |
| Uribe et al. (2025)[16] | N/A | GenAI | GenAI integration in dental education | N/A | Thirty-one guidance documents from 21 universities in 15 countries and three international organizations; dental-specific guidance was essentially absent, prompting recommendations on ethics, prevention of misuse, assessment redesign and the promotion of critical thinking. | Institutional guidance documents rather than empirical studies; rapidly changing policy landscape (search January 2022 – June 2024); no learner outcomes and no language-specific provisions. |
| Rath (2025)[17] | Chinese/English; English-medium oral anatomy | ChatGPT (GPT-4) | Terminology learning and concept explanation in oral anatomy | Pre/post comprehension and engagement; focus groups | Mean comprehension increased by 5.7 points (p<0.001; d=1.91), with positive engagement themes. | Small, single-site uncontrolled pilot; short follow-up and self-report components. |
| Cai et al. (2025)[18] | Not treated as a study variable; the model was developed on dental image–text data in a Chinese clinical setting and reported in English | DentalGPT, 7B multimodal model | Dental visual question answering, disease classification and complex reasoning; comparator: Baseline LLMs | Benchmark performance against baseline models | Demonstrated domain-specific multimodal dental capability. | Preprint; primarily visual/technical; no learner or language-learning outcomes. |
| Firoozi et al. (2025)[19] | N/A | Retrieval-augmented generation (RAG) | Potential RAG applications in dentistry | Narrative synthesis; no original outcome data | Outlined potential for grounding dental LLM output in curated sources. | Narrative/forward-looking evidence; no original educational evaluation. |
| Wang Q. et al. (2026)[20] | Multiple | LLMs (various) | Medical competence evaluation benchmarks | N/A | Static text-based benchmarks dominate | Review of 26 benchmarks identified from 131 records; benchmarks are predominantly static, text-based and examination-derived, with little coverage of interactive, multimodal or cross-lingual clinical communication. |
| Zhu et al. (2025)[21] | English and Chinese | 14 LLMs including DeepSeek-R1, GPT-4o and Claude; DentalCorpus 337.35 million tokens | Bilingual dental question answering (DentalBench); comparator: Across models and languages | Accuracy across tasks, models and languages | Performance varied across models and language/task alignment. | Preprint; public/static benchmark; contamination risk; no learners. |
| Jones et al. (2025)[22] | English | AI chatbot acting as patient and supervisor | Dental-pain history taking and peer-supported virtual-patient role play | Usability, authenticity and learner perceptions | Most respondents perceived the role play as authentic; implementation cost was low. | Small convenience sample; perception-level outcomes; no comparator or clinical transfer. |
| Katonai et al. (2025)[23] | N/A | AI (broad) | AI in primary care | N/A | One hundred and seven studies; AI applications clustered around administrative tasks, medication management and screening/diagnosis, with the greatest potential where tools align with clinical reasoning, workflow and relational care. | Primary care rather than dental education; heterogeneous study designs; usability, training and workflow-integration barriers persist; no language-learning outcomes. |
| Liu et al. (2025)[24] | Mandarin Chinese (local OSCE setting in Taiwan); language was not evaluated as a variable | Low-cost neck-mounted smartphone recording; no AI-language technology | First-person OSCE communication/clinical-skills recording and feedback | Feasibility and video-based assessment/feedback | Demonstrated a low-cost first-person recording approach for OSCE review. | Interns rather than undergraduates; no AI-language intervention. |
| Lopez-Garcia et al. (2026)[25] | English | GPT-4o (Microsoft Azure OpenAI API) with rubric-based and rubric + chain-of-thought prompting; fine-tuned RoBERTa-large and PubMedBERT-large baselines | Automated scoring of physician risk communication; comparator: Human raters | Micro-averaged F1 for 0–5 rubric scores across five shared-decision-making domains, benchmarked against pre-adjudication inter-annotator agreement (F1 83.0–85.4) | Rubric + chain-of-thought prompting with few-shot examples achieved micro-averaged F1 of 85.0–92.0, outperforming the fine-tuned baselines and matching expert inter-annotator agreement. | Twenty single-center oncology consultations; physician–patient encounters rather than students; depends on an upstream sentence-selection model; English only; no educational outcome. |
| Suárez et al. (2022)[26] | Spanish (Dialogflow-based chatbot deployed in a Spanish-language dental curriculum) | AI-chatbot virtual patient | Diagnostic interview and virtual-patient communication practice | Satisfaction, perceived learning and usability | High student satisfaction (reported mean 4.36/5). | Single-site, uncontrolled and perception-dominant; no clinical transfer. |
| Uribe et al. (2024)[27] | N/A | AI chatbots and LLMs | Educator use, perceptions and governance needs | Survey proportions and attitudes | 31% reported AI use and 64% perceived potential for dental education; respondents also raised governance concerns. | Self-selection and self-report; educator rather than learner population. |
| Wang L. et al. (2024)[28] | Chinese | GPT-4o | Automated evaluation of resuscitation-skills examinations; comparator: Human examiners | Section- and total-score agreement with six certified examiners (three junior, three senior) across 103 examinees, plus expert 5-point ratings of GPT-4o reliability | GPT-4o showed moderate agreement with the six experts, and its scoring accuracy was closer to that of senior than of junior examiners. | Video-based CPR skills examination rather than communication; single-center and non-dental; agreement varied markedly by item type. |
| Zouakia et al. (2026)[29] | French | GPT-4o: standard, personalized and simulated-agent configurations | Generation of digital-health OSCE stations; comparator: across the three GPT-4o configurations, with format compliance rated by one expert and educational content rated independently by two digital-health experts blinded to configuration | Expert-rated accuracy, clarity, format and usability | Simulated-agent configuration performed best; 14/16 reviewed stations were usable (88%). | Small expert review; generation study rather than learner evaluation; no clinical transfer. |
| Bannister et al. (2024)[30] | N/A | Generative AI (general) | Academic-integrity policy implications for international students | Policy themes and explicit provisions | Policies provided limited explicit support for international-student circumstances. | Policy sample and jurisdictions may not generalize; no learner outcomes. |
| Kofinas et al. (2025)[31] | N/A | GenAI (general) | Authentic-assessment integrity | Marker ability to detect GenAI input, marks awarded, and changes in the marking and moderation process | Markers were generally unable to distinguish GenAI-assisted from unassisted assessments, although the possibility of GenAI use altered how they marked; authentic assessment alone did not safeguard academic integrity. | Small exploratory sample; single institution and business-education context; no dental, clinical or language-specific findings. |
| Sterling et al. (2025)[32] | English as an additional language (international students in an Australian university) | GenAI writing support (ChatGPT and Claude) | Academic-writing improvement | Appraisal of student writing produced through a series of structured exercises, two of which were repeated with a second, independent student group | Structured, staged use of Gen-AI helped non-native English speakers improve clarity and efficiency, but the authors emphasise that students must retain and develop their own distinct voice rather than delegate it to the tool. | Computing rather than health-professions students; exercise-based rather than controlled design; no validated writing-quality instrument or follow-up. |
| Wetmore et al. (2010)[33] | English | Reflective blogs; no AI | Reflective writing and critical-thinking development | Reflection rubric and Health Sciences Reasoning Test | Blogging improved reflection-rubric performance but not the standardized critical-thinking test. | Pre-AI and dental-hygiene context; transfer to GenAI use is conceptual only. |
| Brewster et al. (2025)[34] | English into Arabic, Armenian, Bengali, simplified Chinese, Somali and Spanish | AI-enabled translation with human-in-the-loop workflow | Discharge-instruction translation workflow evaluation; comparator: ChatGPT-4o alone, professional linguist translation, and human-in-the-loop (AI-generated, linguist post-edited) | Likert ratings (1–5) of linguistic and clinical translation quality by linguists, clinicians and family caregivers; translation time; overall preference | ChatGPT-4o alone performed variably and worst for digitally under-represented languages (Armenian and Somali), whereas human-in-the-loop translations were comparable to or better than professional translations in all six languages, took less time (7.1 vs 16.8 minutes) and were most often preferred. | Paediatric discharge instructions rather than dental education; no learners; expert and caregiver ratings rather than measured patient comprehension or safety outcomes. |
| Amano & Berdejo-Espinola (2025)[35] | Multiple | None (language barriers) | Language barriers in science and conservation | N/A | Sets out three mechanisms by which language barriers impede evidence-based conservation — barriers to generating, synthesizing and applying evidence — and proposes solutions for individuals, institutions, journals, conferences and funders. | Conservation-science perspective article; no health-professions, clinical or educational data. |
| Ross & Stuckler (2025)[36] | English (as foreign language) | None | Barriers to learning English as a foreign language | N/A | Twenty-one of 2,773 screened studies met the criteria; three barrier clusters were identified — foreign-language anxiety (affecting all four language skills, including test anxiety), inhibitory self-beliefs, and linguistic difficulty arising from first-language influence — with substantial overlap between anxiety and negative self-beliefs. | Studies concentrated in the Middle East and Asia with minimal European representation; English-language studies only; general university rather than clinical populations. |
| Al Shamsi et al. (2020)[37] | Multiple | None | Language barriers in healthcare | N/A | Language barriers led to miscommunication, reduced satisfaction for both parties and lower quality and safety of care; interpreter services helped but increased cost and visit length, and one study reported that online translation tools raised satisfaction to 92%. | Only two databases searched (PubMed and Medline); heterogeneous primary studies; patient-care rather than educational setting; predates current GenAI systems. |
| Saigal & Lewis (2023)[38] | N/A | None | Limited English proficiency and linguistic diversity in medicine | N/A | Three teaching cases addressing the appropriate use and loopholes of medical interpreter services, patient–provider language concordance, and the role of medical translation in understanding of disease and medication adherence; argues that interpreter services alone do not remove LEP disparities. | Case-based teaching chapter without primary data; United States context; no educational-outcome measurement. |
| Sliwinski et al. (2024)[39] | N/A | None | LEP outcome disparities and nursing resources | N/A | Patients with limited English proficiency receive lower-quality care and experience worse hospital outcomes; nursing resources — staffing levels and the nurse work environment — are identified as a modifiable factor for reducing these disparities. | Narrative review of hospital nursing outcomes in the United States; no educational, dental or AI component. |
| Khanagar et al. (2026)[40] | Arabic and English | None (language-barrier study; no AI) | Student–patient language barriers in clinical dental care | Survey/interview reports of difficult explanations and communication barriers | Frequently difficult topics included crown lengthening (72.5%) and periodontitis (67.5%). | Single-city, self-report; no AI intervention or comparative outcome. |

**Supplementary Table S4. Included policy and governance documents**

| **Document** | **Issuer** | **Scope** | **Relevant provisions** | **Classification** |
| --- | --- | --- | --- | --- |
| WHO, Ethics and governance of artificial intelligence for health (2021)[1] | World Health Organization | Global; AI for health | Human oversight, transparency, accountability, inclusiveness and equity relevant to clinical education. | Contextual governance |
| UNESCO, Guidance for generative AI in education and research[2] | UNESCO | Global; education/research | Human-centered educational use, integrity, capacity building and age/appropriateness safeguards. | Contextual governance |
| Artificial Intelligence Act, Regulation (EU) 2024/1689[3] | European Union | EU horizontal AI regulation | Risk-based obligations; education/vocational-training systems can fall within regulated high-risk contexts depending on intended use; transparency and literacy duties are relevant. | Contextual governance |
| Generative AI and the EUDPR (2025)[4] | European Data Protection Supervisor | EU institutions; data protection | Lawful basis, data minimization, accountability and safeguards relevant to student/patient data. | Contextual governance |

**Supplementary Table S5. Methodological-reporting and limitations chart**

Y = reported/present; NR = not reported in the source publication or its supplementary materials, or not verifiable from the accessible published record; N/A = not applicable. NR must not be interpreted as ‘No’. TRIPOD-LLM-informed items apply only to LLM performance evaluations and not indiscriminately to NMT studies, qualitative research, educational interventions or policy documents.

| **Source** | **Design** | **Comparator** | **LLM reporting (model/prompt/runs/date)** | **Authentic learner setting** | **Key limitation** |
| --- | --- | --- | --- | --- | --- |
| Hamad et al. (2025)[5] | Systematic review | N/A | N/A | No/indirect | Review-level and non-dental evidence; no AI intervention. |
| Bittle & El-Gayar (2025) [6] | Systematic review | N/A (systematic review) | N/A (no primary model evaluation) | No/indirect | Review-level, non-dental evidence; last search May 2024; no learner outcomes. |
| Strasser et al. (2026)[7] | Mixed-methods benchmarking | Yes (across models, prompt languages and question languages) | Models and versions reported (OpenAI, Meta, Anthropic and DeepSeek models; GPT-4o and Claude Sonnet 3.7 for the qualitative phase); web-interface access dates reported (14 Jan 2025 and 17 Mar 2025); prompt language systematically varied; run count and generation settings NR. | No/indirect | No learners; public-item benchmark; exact prompts/runs require full-text checking. |
| Brewster et al. (2024)[8] | Comparative translation benchmark | Yes | Model reported (ChatGPT-4.0) alongside Google Translate; prompt, run count, access date and generation settings NR. | No/indirect | Small corpus; pediatric and non-dental context; no educational outcomes. |
| Haupt et al. (2025)[9] | Randomized controlled study | Yes | Model reported (GPT-4o); prompts were student-generated and rated by two calibrated examiners rather than standardized; single interaction per question; access date and generation settings NR. | Yes | RCT; two calibrated examiners; short-term knowledge outcome; model/access/prompt detail still requires source-level confirmation. |
| Hendy et al. (2023)[10] | Benchmarking (preprint) | Yes | Models reported (text-davinci-002, text-davinci-003/GPT-3.5, ChatGPT); prompting strategies systematically varied and documented; run count, access date and generation settings NR. | No/indirect | Preprint at the review date; no learner population. |
| Kong et al. (2026)[11] | Comparative accuracy/safety evaluation | Yes | Models reported (ChatGPT-4 and Google Translate); prompt, run count, access date and generation settings NR. | No/indirect | Three target languages; emergency-department instructions; no learners; harm coded from professional back-translations rather than patient comprehension. |
| Noll et al. (2025)[12] | Comparative terminology benchmark | Yes | Models reported (GPT-3.5 and DeepL); prompt, run count, access date and generation settings NR. | No/indirect | Terminology-only corpus; no dental learners or educational outcomes. |
| Sun (2024)[13] | Linguistic translation evaluation | Yes | Systems named (ChatGPT and DeepL) without version numbers; prompt, run count, access date and generation settings NR. | No/indirect | Single literary source text; qualitative comparison without automatic metrics; no clinical or educational outcome. |
| Ranathunga et al. (2023)[14] | Survey (methodological review) | N/A | N/A | No/indirect | Methodological survey; no primary evaluation, learner population or educational outcome. |
| El-Hakim et al. (2025)[15] | Scoping review | N/A | N/A | No/indirect | Review-level; six databases searched March 2025; English-language studies only; no language, communication or international-student focus. |
| Uribe et al. (2025)[16] | Scoping review | N/A | N/A | No/indirect | Institutional guidance documents rather than empirical studies; no dental-specific guidance identified; no learner outcomes. |
| Rath (2025)[17] | Convergent mixed-methods pilot; 8 weeks | NR (single-group pre/post pilot; no control condition) | Model reported (ChatGPT, GPT-4); prompt structure, run count, access date and generation settings NR. | Yes | Pre/post pilot with p value and effect size; small, uncontrolled, single-site study. |
| Cai et al. (2025)[18] | Multimodal model development and benchmark (preprint) | Yes (baseline multimodal LLMs on the same benchmark) | Model architecture and size reported (7B multimodal), with training corpus (>120,000 annotated dental images) and staged supervised fine-tuning plus reinforcement learning; prompts, run count, access date and generation settings NR. | No/indirect | Preprint; primarily visual/technical; no learner or language-learning outcomes. Preprint status at review date. |
| Firoozi et al. (2025)[19] | Mini-review | N/A (mini-review) | N/A (no primary model evaluation) | No/indirect | Narrative/forward-looking evidence; no original educational evaluation. |
| Wang Q. et al. (2026)[20] | Review of benchmarks | N/A (review of benchmarks) | N/A (no primary model evaluation) | No/indirect | Twenty-six benchmarks reviewed; predominantly static and text-based, derived from licensing examinations; little coverage of interactive or cross-lingual communication. |
| Zhu et al. (2025)[21] | Bilingual benchmark (preprint) | Yes | Fourteen models named, including DeepSeek-R1, GPT-4o and Claude; corpus size reported (DentalCorpus, 337.35 million tokens; 36,597 bilingual items); prompts, run count, access dates and generation settings NR. | No/indirect | Preprint; public/static benchmark; contamination risk; no learners. Preprint status at review date. |
| Jones et al. (2025)[22] | Classroom implementation/usability study | NR (single-group classroom implementation; no control condition) | Chatbot roles described (patient and supervisor); underlying model, version, prompts, run count and access date NR. | Yes | Usability survey n=14; no comparator; perception-level outcome. |
| Katonai et al. (2025)[23] | Scoping review | N/A | N/A | No/indirect | Primary care rather than education; 107 heterogeneous studies; usability, training and workflow-integration barriers dominate. |
| Liu et al. (2025)[24] | Feasibility/device evaluation | NR (pre/post questionnaire design; no control group) | N/A | Yes | Interns rather than undergraduates; no AI-language intervention. |
| Lopez-Garcia et al. (2026)[25] | LLM evaluation study | Yes (fine-tuned RoBERTa-large and PubMedBERT-large baselines, plus human annotators) | GPT-4o accessed via the Microsoft Azure OpenAI API; rubric-only and rubric + chain-of-thought prompts documented, with 6–10 few-shot examples per domain; temperature 0.3 and top-p 0.4 reported; number of repeated runs and access date NR. | No/indirect | Twenty single-center oncology consultations; no student population; performance depends on an upstream sentence-selection model; English only. |
| Suárez et al. (2022)[26] | Descriptive cross-sectional educational study | NR (single-group descriptive cross-sectional design) | Chatbot platform reported (Dialogflow); underlying model, version, prompts, run count, access date and generation settings NR. | Yes | Large student sample but descriptive/uncontrolled; primarily satisfaction/perception outcomes. |
| Uribe et al. (2024)[27] | Cross-sectional worldwide survey; 31 items (May–June 2023) | N/A (cross-sectional survey) | N/A (survey of educator use and perceptions; no primary model evaluation) | Yes | Self-selection and self-report; educator rather than learner population. |
| Wang L. et al. (2024)[28] | Evaluation study | Yes | Model reported (GPT-4o); prompt, run count, access date and generation settings NR. | No/indirect | Video-based CPR skills examination rather than communication; single-center and non-dental; agreement moderate overall and weaker for depth- and recoil-related items. |
| Zouakia et al. (2026)[29] | Comparative configuration study | Yes | Model reported (GPT-4o) with three documented configurations (standard, personalized, simulated-agents) and their prompt structures and reference material described; run count, access date and generation settings NR. | No/indirect | Two blinded content experts reported; configuration comparison; no learner outcomes. |
| Bannister et al. (2024)[30] | Qualitative document/policy analysis | N/A (qualitative document/policy analysis) | N/A | No/indirect | Policy sample and jurisdictions may not generalize; no learner outcomes. |
| Kofinas et al. (2025)[31] | Exploratory experimental marking and moderation study | Yes (GenAI-assisted versus unassisted assessment scripts) | GenAI use described at task level; model, version, prompts, run count, access date and generation settings NR. | Yes | Small exploratory sample; single institution; business rather than clinical education; no language-specific analysis. |
| Sterling et al. (2025)[32] | Structured exercise-based educational study (two independent student groups) | NR (no control group; two exercises repeated with a second, independent group) | Tools named (ChatGPT and Claude); model versions, prompts, run count, access date and generation settings NR. | Yes | Computing rather than health-professions students; no validated writing-quality measure or follow-up assessment. |
| Wetmore et al. (2010)[33] | Controlled two-group pre/post educational study (pre-AI) | Yes | N/A | Yes | Controlled pre/post design; standardized HSRT plus reflection rubric; pre-AI. |
| Brewster et al. (2025)[34] | Multidisciplinary evaluation | Yes | Model and version reported (ChatGPT-4o, version 2024-11-20); prompts, run count, access date and generation settings NR. | No/indirect | Pediatric discharge instructions; no learner population; rating-based outcomes rather than measured patient comprehension. |
| Amano & Berdejo-Espinola (2025)[35] | Review/perspective article | N/A | N/A | No/indirect | Conservation-science context; no health-professions or educational data. |
| Ross & Stuckler (2025)[36] | Systematic review | N/A | N/A | No/indirect | Twenty-one studies concentrated in the Middle East and Asia; English-language studies only; general university populations. |
| Al Shamsi et al. (2020)[37] | Systematic review | N/A | N/A | No/indirect | Only two databases searched; heterogeneous primary studies; patient-care rather than educational setting; predates current GenAI systems. |
| Saigal & Lewis (2023)[38] | Book chapter (three teaching cases) | N/A (case-based chapter) | N/A | No/indirect | No primary data; United States context; no educational-outcome measurement. |
| Sliwinski et al. (2024)[39] | Review | N/A | N/A | No/indirect | Narrative review of hospital nursing outcomes in the United States; no AI or educational component. |
| Khanagar et al. (2026)[40] | Mixed-methods study | NR (descriptive mixed-methods design) | N/A | Yes | Mixed methods with students and patients; single-city self-report; no AI intervention. |

**Supplementary Table S6. Operationalization of the data-charting framework**

| **Charting category** | **Operational meaning** | **Use in synthesis** |
| --- | --- | --- |
| **Publication and source characteristics** | Author, year, country/setting, source type, empirical or policy/guidance status. | Used to describe the evidence base and distinguish non-policy sources from policy or grey literature (Supplementary Tables S3–S4) |
| **Educational or clinical context** | Dental education, medical education, health professions education, patient safety or governance context. | Used, together with Table 3, to determine transferability and directness of relevance |
| **Target learner or user group** | International students, students with LEP, multilingual learners, dental students, health professions students, educators or patients (per the definitions in Table 2). | Used to identify whether findings applied directly or indirectly to the review population |
| **Technology type and technical characteristics** | GenAI, LLMs, GPT-based chatbots, NMT or related language technology, including the technical-reporting items in Section 2.7 (model, version, prompting, RAG, fine-tuning, etc.). | Used to avoid treating all AI tools as a single undifferentiated category and to support Supplementary Tables S3 and S5 |
| **Language or communication function** | Terminology translation, multilingual tutoring, OSCE or virtual-patient communication practice, or reflective writing support, mapped to the task contexts in Table 2. | Used to generate the four application domains reported in the Results. |
| **Reported outcome and outcome level** | Outcome measures classified by the six outcome levels defined in Section 2.8. | Used to summarize potential benefits without conflating technical performance with educational effectiveness. |
| **Methodological limitations** | The items listed in Section 2.7 (sample size, comparator, validation, evaluators, blinding, model runs, prompt and version disclosure, etc.). | Used to qualify the strength of the evidence (Section 3.6; Supplementary Table S5). |
| **Risks and implementation issues** | Mistranslation, hallucination, over-reliance, academic-integrity concerns, privacy, bias or inequitable access. | Used to inform the discussion of the cross-cutting governance theme and the implementation pathway. |

**Supplementary Table S7. Learner and institutional factors**

| **Factor** | **Description** | **Implication for AI-enabled language support** |
| --- | --- | --- |
| **Language distance from the instructional language** | The learner’s first language may be linguistically close to or distant from the language of instruction. | Students with greater language distance may require contextual translation, bilingual glossaries and carefully checked terminology support. |
| **Prior clinical exposure** | Students may enter the host program with different levels of clinical observation or patient-contact experience. | Students with limited exposure may benefit from scaffolded case explanations and low-stakes virtual-patient practice. |
| **Technology access and literacy** | Students differ in access to digital tools and in familiarity with prompting, translation software and learning platforms. | Institutions should provide approved tools and basic training rather than leaving students to rely on unregulated consumer systems. |
| **Cultural distance** | Norms for authority, pain expression, disclosure, consent and patient participation vary across cultures. | Virtual-patient scenarios can be designed to rehearse culturally appropriate patient communication in the host setting. |
| **Institutional support** | Schools differ in the availability of language support, bilingual glossaries, translation services and faculty awareness. | In lower-support settings, institutionally validated tools may reduce inequity and improve consistency of support. |

**Supplementary Table S8. PRISMA-ScR checklist**

| **Item** | **Topic** | **PRISMA-ScR requirement** | **Location in manuscript** |
| --- | --- | --- | --- |
| 1 | Title | Identify the report as a scoping review. | p. 1; Title |
| 2 | Structured summary | Provide background, objectives, methods, results and conclusions. | p. 2; Abstract |
| 3 | Rationale | Describe rationale in context of existing knowledge. | p. 5; Introduction |
| 4 | Objectives | State questions/objectives using key PCC elements. | p. 5; Introduction, final paragraph |
| 5 | Protocol/registration | State whether a protocol exists and registration information. | p. 6; Section 2.1; no a priori protocol/registration |
| 6 | Eligibility criteria | Specify characteristics and rationale. | p. 6; Section 2.3; Tables 1–3 |
| 7 | Information sources | Describe databases, dates and other sources. | p. 6; Section 2.4; Supplementary S1–S2 |
| 8 | Search | Present a reproducible search strategy for at least one database. | p. 6; Section 2.4; Supplementary Table S1 (full PubMed, Scopus and Web of Science strings) |
| 9 | Selection of evidence | State screening and eligibility process. | p. 6; Section 2.5 |
| 10 | Data charting | Describe charting methods and calibration/duplication. | p. 6; Section 2.6; Supplementary S6 |
| 11 | Data items | List and define variables sought. | p. 6; Sections 2.6–2.7; Supplementary S5–S6 |
| 12 | Critical appraisal | Describe appraisal, if conducted, and its use. | p. 6; Section 2.7; no scored appraisal; descriptive charting |
| 13 | Synthesis | Describe handling and synthesis methods. | p. 6; Section 2.8 |
| 14 | Selection results | Report numbers and reasons, ideally with flow diagram. | p. 13; Section 3.1; Figure 2; Supplementary S2B |
| 15 | Source characteristics | Present characteristics and citations. | p. 13; Section 3.3; Supplementary S3–S4 |
| 16 | Critical appraisal results | Present appraisal results, if performed. | p. 13; Section 3.6; Supplementary S5 |
| 17 | Individual-source results | Present relevant charted data for each source. | p. 13; Supplementary S3–S5 |
| 18 | Synthesis of results | Summarize charting linked to questions/objectives. | p. 13; Sections 3.2–3.6; Tables 4–5 |
| 19 | Summary of evidence | Summarize main results, relevance and evidence. | p. 20; Sections 4.1–4.5 |
| 20 | Limitations | Discuss limitations of process and evidence. | p. 20; Sections 4.6–4.7 |
| 21 | Conclusions | Provide overall interpretation and implications. | p. 24; Section 5 |
| 22 | Funding | Describe funding and funder role. | p. 3; Declarations—Funding |

**References**

1.	Team, W.H.E.G., *Ethics and governance of artificial intelligence for health*. 2021.

2.	UNESCO, *Guidance for generative AI in education and research*. 2023.

3.	European Union, *Laying down harmonised rules on artificial intelligence and amending Regulations (EC) No 300/2008, (EU) No 167/2013, (EU) No 168/2013, (EU) 2018/858, (EU) 2018/1139 and (EU) 2019/2144 and Directives 2014/90/EU, (EU) 2016/797 and (EU) 2020/1828 (Artificial Intelligence Act)*, E. Union, Editor. 2024.

4.	The EU's independent dataprotection authority, *Generative Al and the EUDPR: Orientations forensuring data protectioncompliance when usingGenerative Al systems.* 2025.

5.	Hamad, A.A., et al., *Decolonizing medical education: a systematic review of educational language barriers in countries using foreign languages for instruction.* BMC Med Educ, 2025. **25**(1): p. 701.

6.	Bittle, K. and O. El-Gayar, *Generative AI and academic integrity in higher education: A systematic review and research agenda.* Information, 2025. **16**(4): p. 296.

7.	Strasser, L.M., et al., *Performance Evaluation of Large Language Models in Multilingual Medical Multiple-Choice Questions: Mixed Methods Study.* JMIR Med Educ, 2026. **12**(1): p. e81399.

8.	Brewster, R.C.L., et al., *Performance of ChatGPT and Google Translate for Pediatric Discharge Instruction Translation.* Pediatrics, 2024. **154**(1): p. e2023065573.

9.	Haupt, F., T. Rodig, and P. Liersch, *Evaluating ChatGPT-4o as an Educational Support Tool for the Emergency Management of Dental Trauma: Randomized Controlled Study Among Students.* JMIR Med Educ, 2025. **11**(1): p. e80576.

10.	Hendy, A., et al., *How good are gpt models at machine translation? A comprehensive evaluation. arXiv 2023.* arXiv preprint arXiv:2302.09210, 2023.

11.	Kong, M., et al., *Evaluation of the accuracy and safety of machine translation of patient-specific discharge instructions: a comparative analysis.* BMJ Qual Saf, 2026. **35**(3): p. 150–158.

12.	Noll, R., et al., *Assessing GPT and DeepL for terminology translation in the medical domain: A comparative study on the human phenotype ontology.* BMC Med Inform Decis Mak, 2025. **25**(1): p. 237.

13.	Sun, R., *Evaluating the translation accuracy of ChatGPT and DeepL through the lens of implied subjects.* AWEJ for Translation & Literary Studies, 2024. **8**(4).

14.	Ranathunga, S., et al., *Neural machine translation for low-resource languages: A survey.* ACM Computing Surveys, 2023. **55**(11): p. 1–37.

15.	El-Hakim, M., R. Anthonappa, and A. Fawzy, *Artificial Intelligence in Dental Education: A Scoping Review of Applications, Challenges, and Gaps.* Dent J (Basel), 2025. **13**(9): p. 384.

16.	Uribe, S.E., I. Maldupa, and F. Schwendicke, *Integrating Generative AI in Dental Education: A Scoping Review of Current Practices and Recommendations.* Eur J Dent Educ, 2025. **29**(2): p. 341–355.

17.	Rath, A., *Leveraging ChatGPT to support terminology learning in oral anatomy: a mixed-methods study among linguistically diverse dental students.* BMC Med Educ, 2025. **25**(1): p. 1425.

18.	Cai, Z., et al., *DentalGPT: Incentivizing Multimodal Complex Reasoning in Dentistry.* arXiv preprint arXiv:2512.11558, 2025.

19.	Firoozi, T., R. Adabdokht, and H. Lai, *Retrieval augmented generation in dentistry: potentials, applications, and future directions.* Front Dent Med, 2025. **6**: p. 1760990.

20.	Wang, Q., et al., *A Survey on Medical Competence Evaluation Benchmarks for Large Language Models.* Health Care Sci, 2026. **5**(1): p. 4–18.

21.	Zhu, H., et al., *DentalBench: benchmarking and advancing LLMs capability for bilingual dentistry understanding.* arXiv preprint arXiv:2508.20416, 2025.

22.	Jones, B., A. Desu, and C.D.F. Honig, *Artificial Intelligence Chatbots as Virtual Patients in Dental Education: A Constructivist Approach to Classroom Implementation.* Eur J Dent Educ, 2026. **30**(2): p. 447–456.

23.	Katonai, G., N. Arvai, and B. Mesko, *AI and Primary Care: Scoping Review.* J Med Internet Res, 2025. **27**: p. e65950.

24.	Liu, Y.C., et al., *Evaluating communication and clinical skills in objective structured clinical examination (OSCE) using a low-cost neck-mounted device, insights from a first-person perspective.* J Dent Sci, 2025. **20**(4): p. 2436–2442.

25.	Lopez-Garcia, G., et al. *Scoring Physician Risk Communication in Prostate Cancer Using Large Language Models*. in *Biocomputing 2026: Proceedings of the Pacific Symposium*. 2025. World Scientific.

26.	Suarez, A., et al., *Using a Virtual Patient via an Artificial Intelligence Chatbot to Develop Dental Students' Diagnostic Skills.* Int J Environ Res Public Health, 2022. **19**(14): p. 8735.

27.	Uribe, S.E., et al., *Artificial intelligence chatbots and large language models in dental education: Worldwide survey of educators.* Eur J Dent Educ, 2024. **28**(4): p. 865–876.

28.	Wang, L., et al., *Suitability of GPT-4o as an evaluator of cardiopulmonary resuscitation skills examinations.* Resuscitation, 2024. **204**: p. 110404.

29.	Zouakia, Z., et al., *AI-Driven Objective Structured Clinical Examination Generation in Digital Health Education: Comparative Analysis of Three GPT-4o Configurations.* JMIR Med Educ, 2026. **12**(1): p. e82116.

30.	Bannister, P., E.A. Peñalver, and A.S. Urbieta, *International students and generative artificial intelligence: A cross-cultural exploration of HE academic integrity policy.* Journal of international students, 2024. **14**(3): p. 149–170.

31.	Kofinas, A.K., C.H.H. Tsay, and D. Pike, *The impact of generative AI on academic integrity of authentic assessments within a higher education context.* British Journal of Educational Technology, 2025. **56**(6): p. 2522–2549.

32.	Sterling, L., et al., *Finding your voice: Using generative ai to help international students improve their writing.* Information, 2025. **16**(4): p. 289.

33.	Wetmore, A.O., et al., *Reflective blogs in clinical education to promote critical thinking in dental hygiene students.* J Dent Educ, 2010. **74**(12): p. 1337–50.

34.	Brewster, R.C., et al., *Evaluating human-in-the-loop strategies for artificial intelligence-enabled translation of patient discharge instructions: a multidisciplinary analysis.* NPJ Digit Med, 2025. **8**(1): p. 629.

35.	Amano, T. and V. Berdejo-Espinola, *Language barriers in conservation: consequences and solutions.* Trends Ecol Evol, 2025. **40**(3): p. 273–285.

36.	Ross, B. and D. Stuckler, *Barriers to learning English as a foreign language (EFL) for university students: A systematic review.* International Journal of Instruction, 2025. **18**(4): p. 395–420.

37.	Al Shamsi, H., et al., *Implications of Language Barriers for Healthcare: A Systematic Review.* Oman Med J, 2020. **35**(2): p. e122.

38.	Saigal, T. and K. Lewis, *Healthcare in patients with limited English proficiency and linguistic diversity in medicine*, in *Cases on Diversity, Equity, and Inclusion for the Health Professions Educator*. 2023, IGI Global Scientific Publishing. p. 130–148.

39.	Sliwinski, K., et al., *A Review of Disparities in Outcomes of Hospitalized Patients with Limited English Proficiency: The Importance of Nursing Resources.* J Health Care Poor Underserved, 2024. **35**(1): p. 359–374.

40.	Khanagar, S.B., et al., *Assessment of Language Barriers Between Dental Students and Patients in Riyadh, Saudi Arabia-A Mixed Methods Study.* Dent J (Basel), 2026. **14**(2): p. 115.
